# Diabetic Neuropathic Arthropathy of the Knee: Two Case Reports and a Review of the Literature

**DOI:** 10.1155/2018/9301496

**Published:** 2018-01-23

**Authors:** Amit Patel, Aaron K. Saini, Michael E. Edmonds, Venu Kavarthapu

**Affiliations:** ^1^Department of Trauma and Orthopaedics, King's College Hospital NHS Foundation Trust, Denmark Hill, London SE5 9RS, UK; ^2^Diabetic Foot Clinic, King's College Hospital NHS Foundation Trust, Denmark Hill, London SE5 9RS, UK

## Abstract

Diabetic neuroarthropathy of the knee or Charcot knee (CK) is a lesser-known complication of diabetes mellitus, with a limited number of case reports and small case series published in the literature. The majority of these reports describe the complexities and challenges that arise in these patients undergoing knee arthroplasty procedures. We present two cases of CK, including a rare case of concurrent bilateral disease, and also a review of the current literature.

## 1. Introduction

Charcot disease of the foot is a well-described complication of diabetes mellitus and other neuropathic disease processes. Similar pathology can also occur in the knee, but this remains an underreported and underresearched area [1]. It should be considered in a patient with peripheral neuropathy, who presents with a red, hot, tender, and swollen knee joint. We describe here two cases of CK and review the literature available.

## 2. Case 1

A 44-year-old gentleman, who had been under our care for the management of a plantar ulcer on his right forefoot, presented to our multidisciplinary diabetic foot clinic with recent onset of pain and swelling in the right knee. He had been diagnosed with Type 1 diabetes at the age of 8 and had an established peripheral neuropathy. He had a past medical history of hypertension. He gave a three-month history of right knee pain, along with gradually worsening knee swelling. The pain was throbbing in nature, triggered by weight bearing, but also felt at night. There was no history of trauma to the knee.

On examination, his knee was warm to touch with no effusion. There was tenderness over the superolateral aspect of the patella and the lateral femoral condyle. Clinically his knee had a full range of motion and was stable.

**Figure 1:**
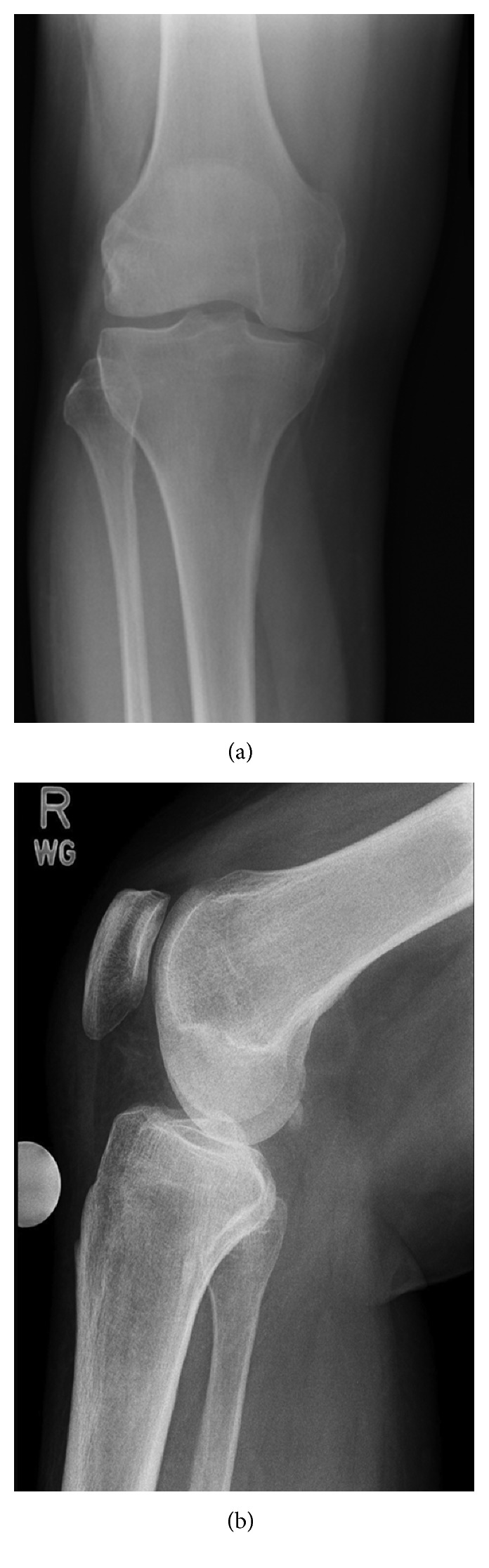
Initial (a) AP radiograph of the knee and (b) Lateral radiograph of the knee were normal.

**Figure 2:**
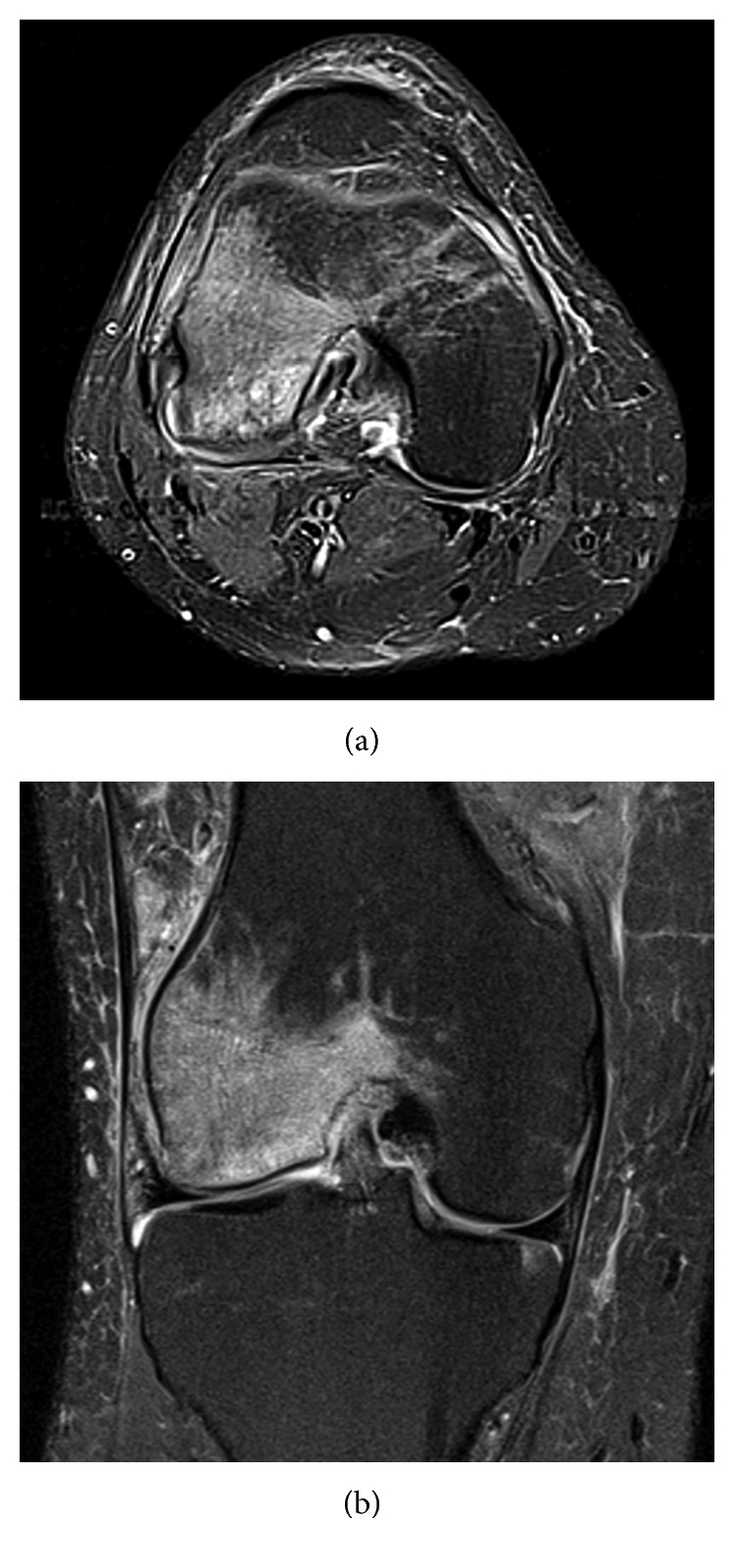
MRI (T2 Fat Sat; (a) axial and (b) coronal views) showing lateral and medial femoral bone oedema (osteochondral collapse of lateral femoral condyle).

**Figure 3:**
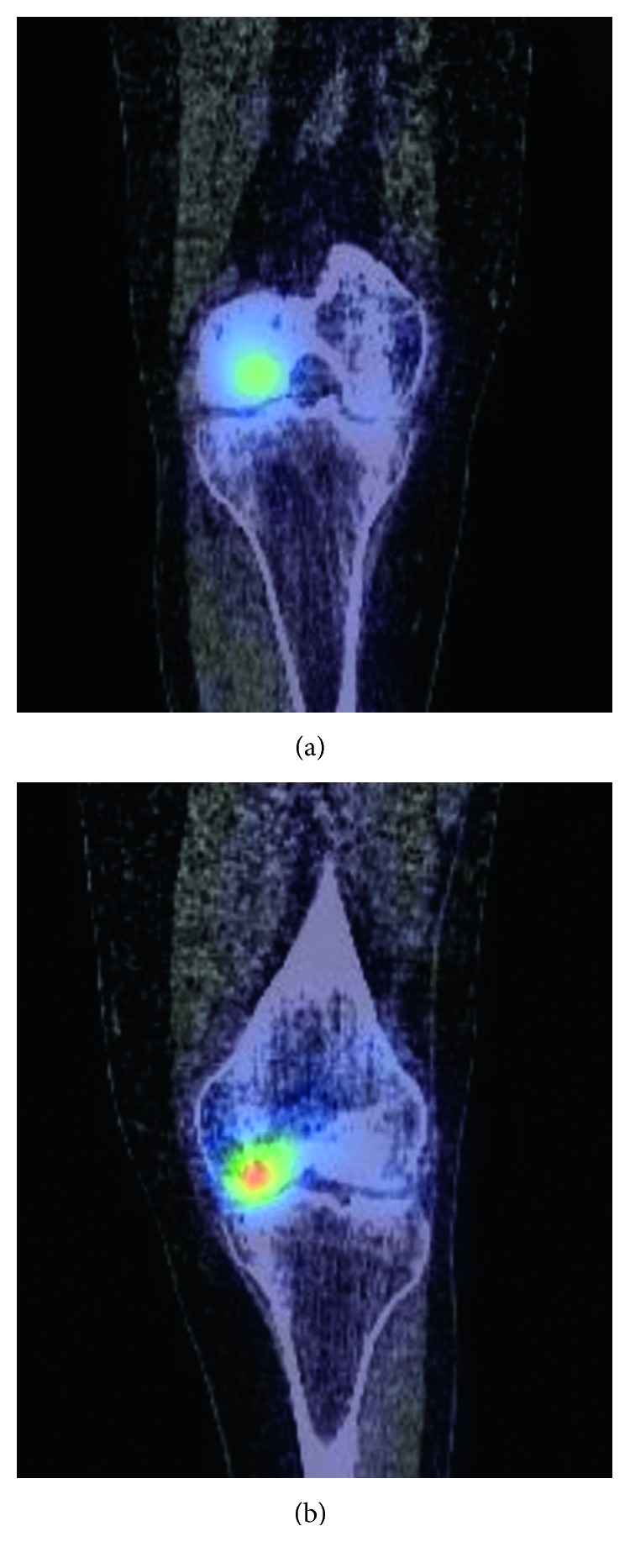
(a) and (b) Sagittal CT component of SPECT showing increased focal tracer uptake in the left medial femoral condyle.

Initial plain radiographs of the knee did not show any obvious abnormality (Figure 1), and he was placed in a knee brace, recommended non-weight bearing, pending further investigation. Magnetic resonance imaging (MRI) (Figure 2) suggested multiple areas of osteonecrosis in the subchondral portion of distal femur. Single positron emission computed tomography (SPECT) imaging (Figure 3) confirmed the diagnosis of CK with increased tracer update, associated with increased vascularity in the medial and to a greater degree the lateral femoral condyles. Computed tomography (CT) component of the scan also demonstrated medullary infarcts in the proximal right femur.

He was managed with non-weight bearing in a hinged knee brace, initially fixed in extension for six weeks, followed by gradal return to full weight bearing with the knee brace on and gradual increase in flexion range over a 3-month period to a good clinical effect.

**Figure 4:**
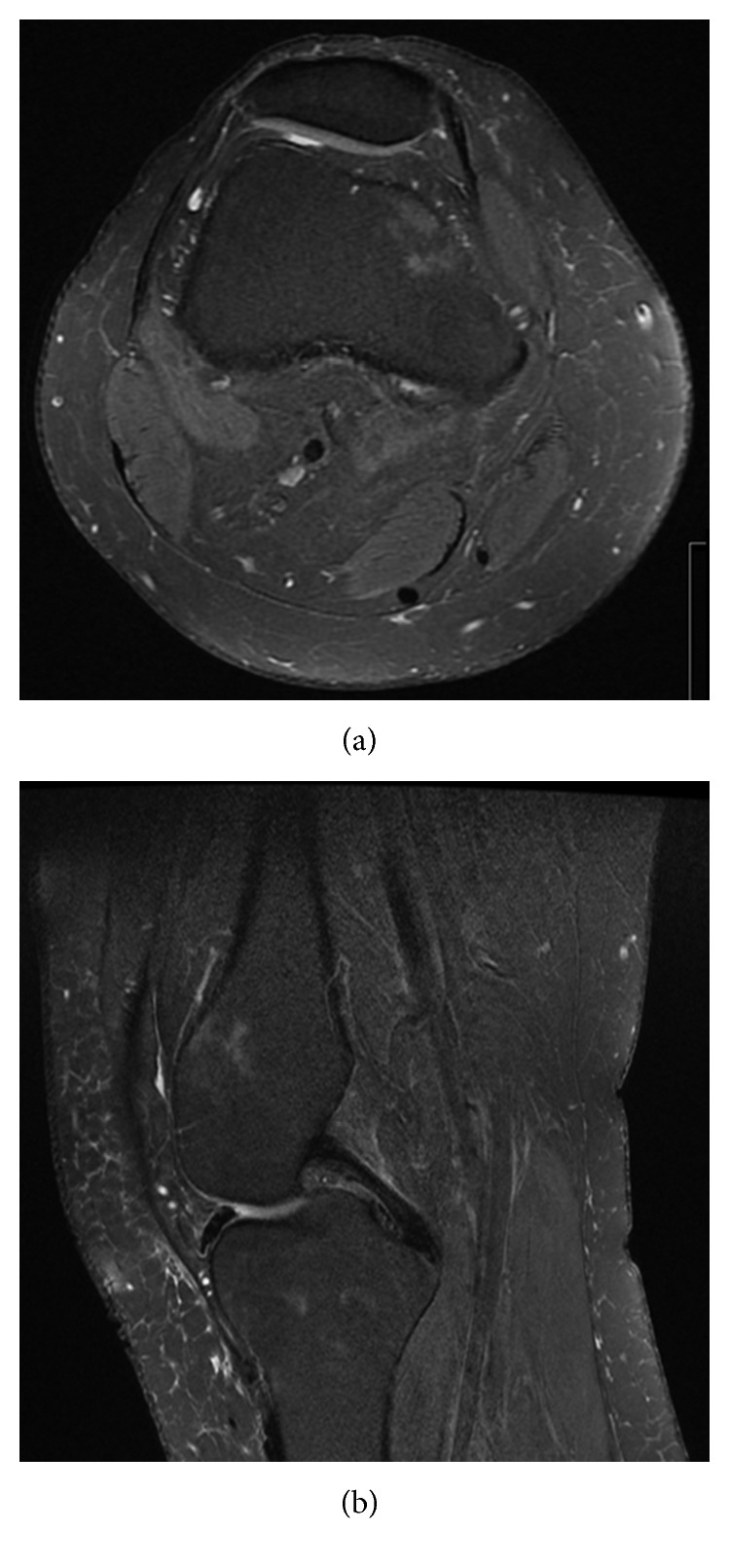
MRI (T2 Fat Sat; (a) axial and (b) sagittal images of left knee) showing medial femoral condyle marrow oedema.

**Figure 5:**
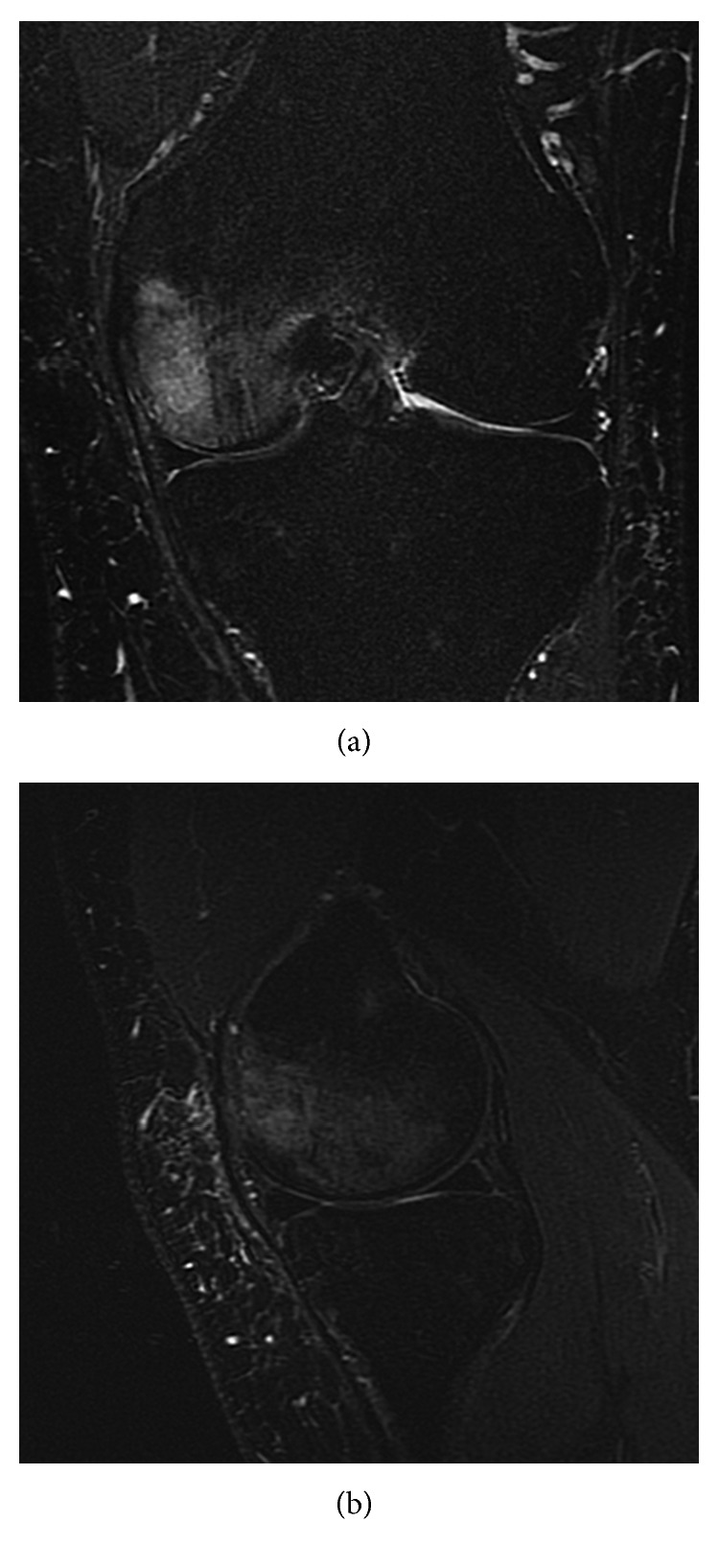
(a) Coronal and (b) sagittal images of right knee showing lateral femoral condyle marrow oedema.

Six months after his initial presentation, he re-presented with bilateral knee symptoms and similar MRI changes (Figures 4 and 5). Plain radiographs again did not show any significant pathology. Nonoperative management again with non-weight bearing (wheelchair use) and a hinged knee brace was applied. At six months, he had progressed to full weight bearing on the right knee with a 0–90 degrees range of motion and partial weight bearing on the left-hand side with a 0–90 degree range of motion. Thirteen months later, he had full radiological and clinical resolution.

Four years later (six years after initial presentation), he attended the diabetic foot clinic with right foot pain and right knee pain. He had a pedal temperature difference of 2 degrees on the right foot and 0.5 degree on the right knee. He was placed in a total contact cast for the foot with a clinical diagnosis of acute Charcot neuroarthropathy. An MRI of the right knee was performed which demonstrated minor bone oedema to the medal femoral condyle in keeping with a third episode of CK in the right knee. This clinically settled quite quickly, most likely with the non-weight-bearing nature of the Charcot foot on the concurrent side. Within six months, he redeveloped symptoms in the left knee which were managed with touch weight bearing and a hinged knee brace for four weeks (0–60 degrees) followed by progression to full weight bearing over the course of the next three months.

## 3. Case 2

A 46-year-old lady was referred to our multidisciplinary unit with a two-week history of atraumatic left knee swelling and pain. She was diagnosed with Type 1 diabetes at the age of 14 and suffered with established peripheral neuropathy and nephropathy. She was a smoker. She had previously suffered from Charcot neuroarthropathy of the left foot with recalcitrant ulceration on the plantar aspect and a rocker bottom deformity. She had previously undergone midfoot osteotomy and frame application of this to good effect.

The examination by the orthopaedic surgical team revealed moderate swelling of the left knee, along with local warmth and tenderness over the lateral part of the knee joint. Moderate degree of medial and lateral collateral ligament laxity was also noted.

**Figure 6:**
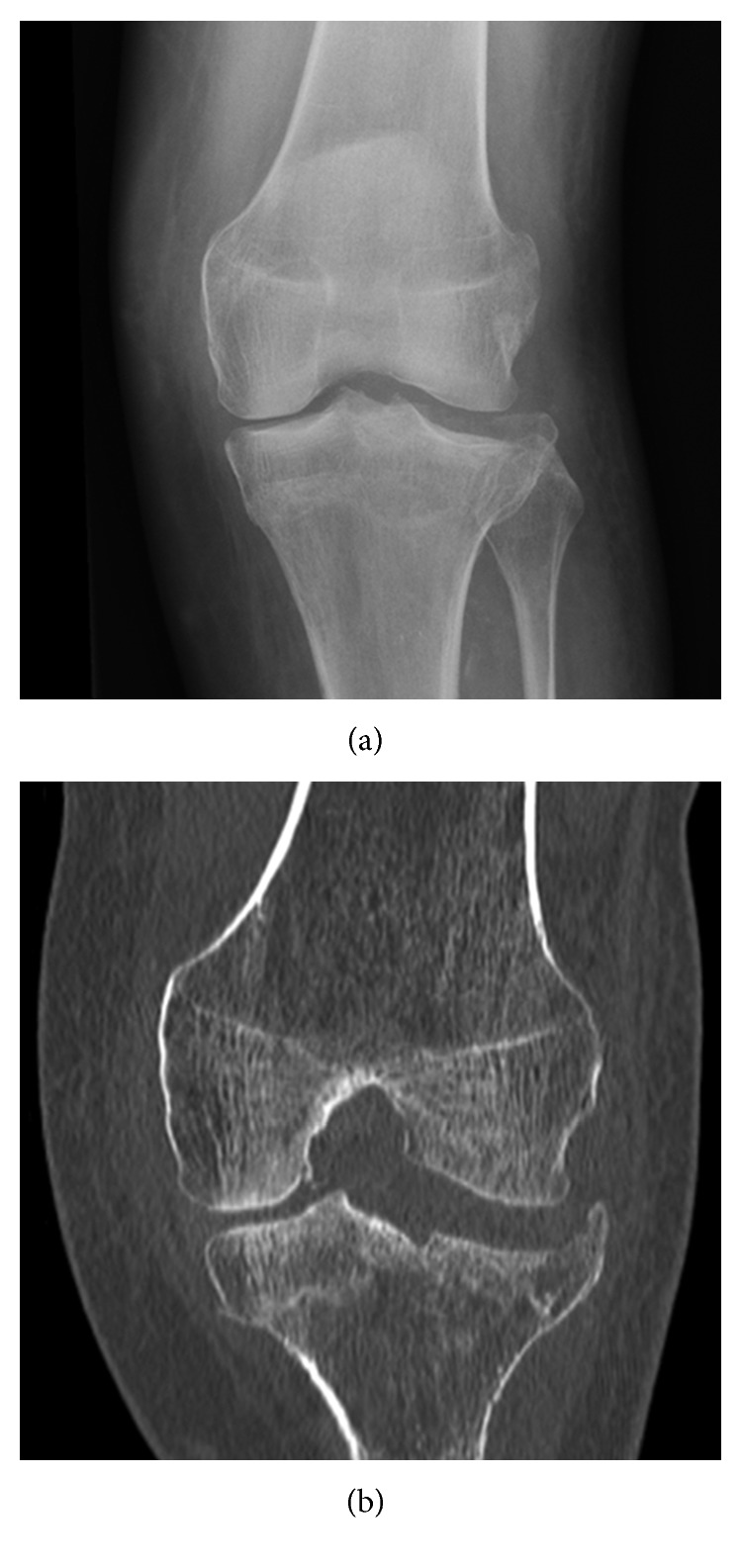
Patient 2 initial presentation. (a) Anterior-posterior radiograph and (b) coronal CT scan.

Plain radiographs and CT of the knee at the time of presentation demonstrated a displaced lateral tibial plateau fracture, with a lateral split and significant depression (Figure 6).

The patient was managed with an above-knee total contact cast and advised to not weight-bear on the limb (wheelchair). It was felt that operative management would be unsuitable due to an ongoing infection of a plantar toe ulcer.

**Figure 7:**
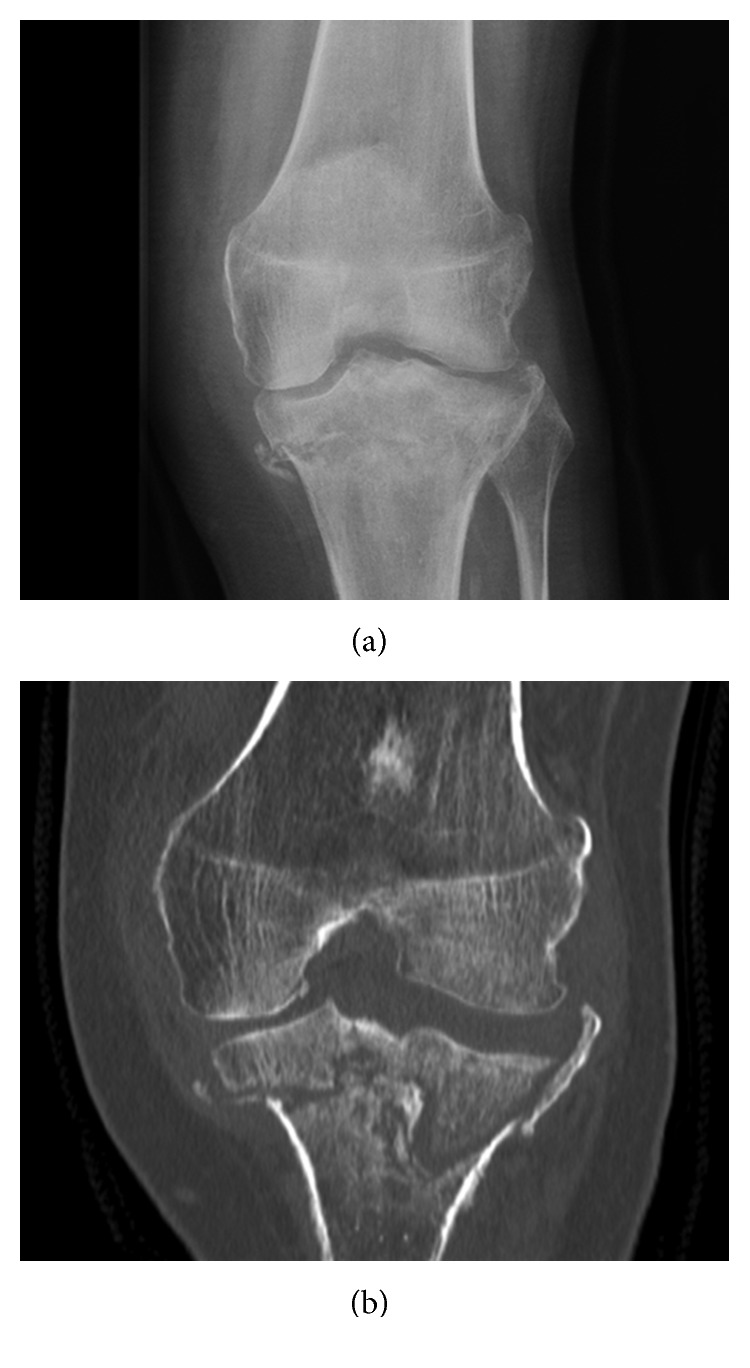
Delayed healing. (a) AP radiograph and (b) coronal CT scan before Exogen treatment.

**Figure 8:**
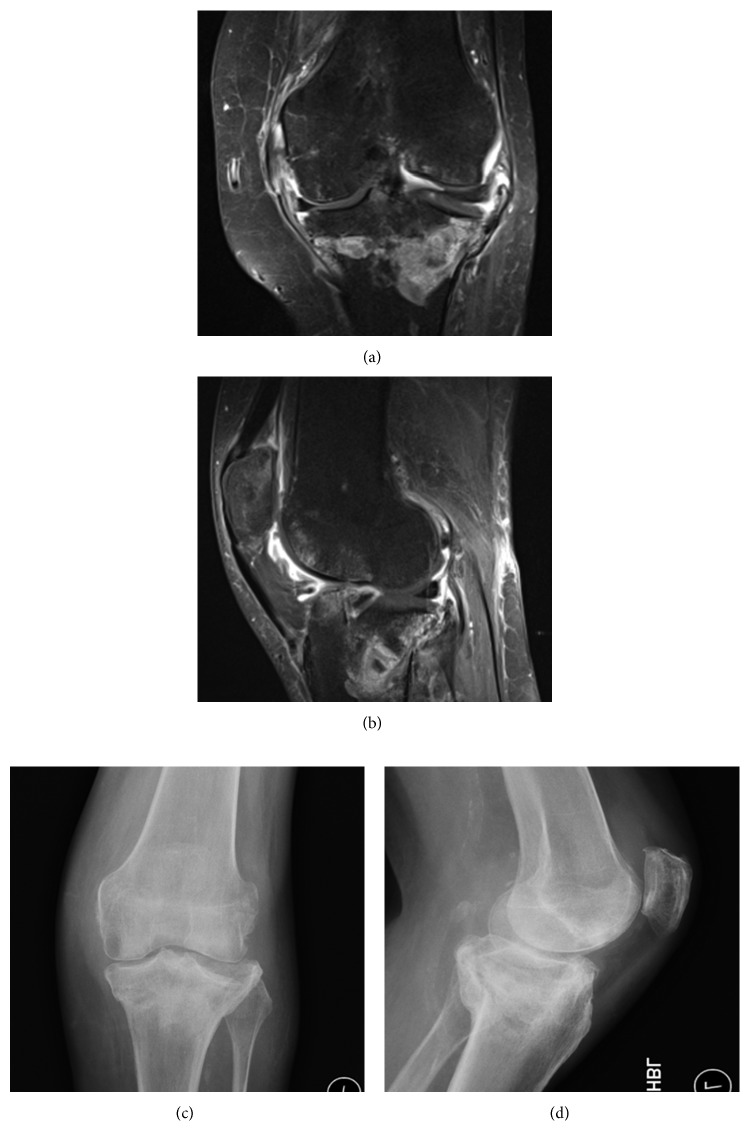
Post-Exogen therapy. (a) Coronal MRI T1 Fat Sat + Gad, (b) sagittal MRI coronal Fat Sat, (c) AP, and (d) lateral radiographs.

Low-intensity pulsed ultrasound therapy (Exogen, Bioventus LLC) was commenced four months after presentation due to a lack of radiological evidence of bone healing (Figure 7). Total contact casting was converted to a hinged knee brace locked in extension at five months after initial treatment due to contact ulceration on the anterior tibia from the cast. Low-intensity pulsed ultrasound therapy was stopped after a three-month treatment as the radiographs showed good evidence of bone healing (Figure 8). The knee bracing was continued for further three months during which time she was allowed to gradually return to full weight bearing and increased knee range of motion.

**Figure 9:**
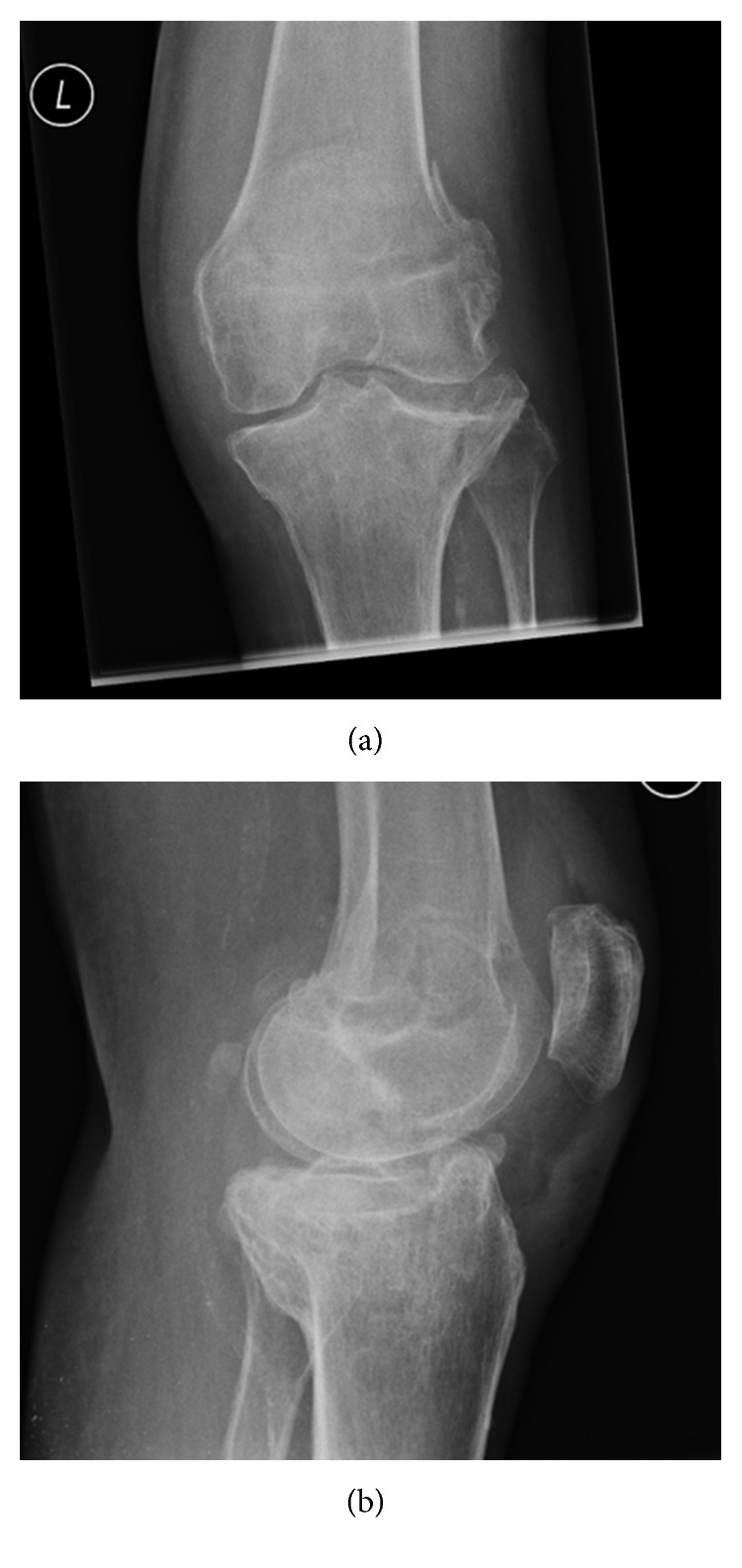
(a) AP and (b) lateral radiographs demonstrating intra-articular femoral and tibial fractures.

Four years after her initial presentation, she was admitted with left knee pain. On examination, her left knee was clinically warm and erythematous with deformity. Plain radiographs demonstrated an atraumatic left femoral fracture and an intra-articular fracture of the lateral tibial plateau. She was managed in a long leg cast (Figure 9).

**Figure 10:**
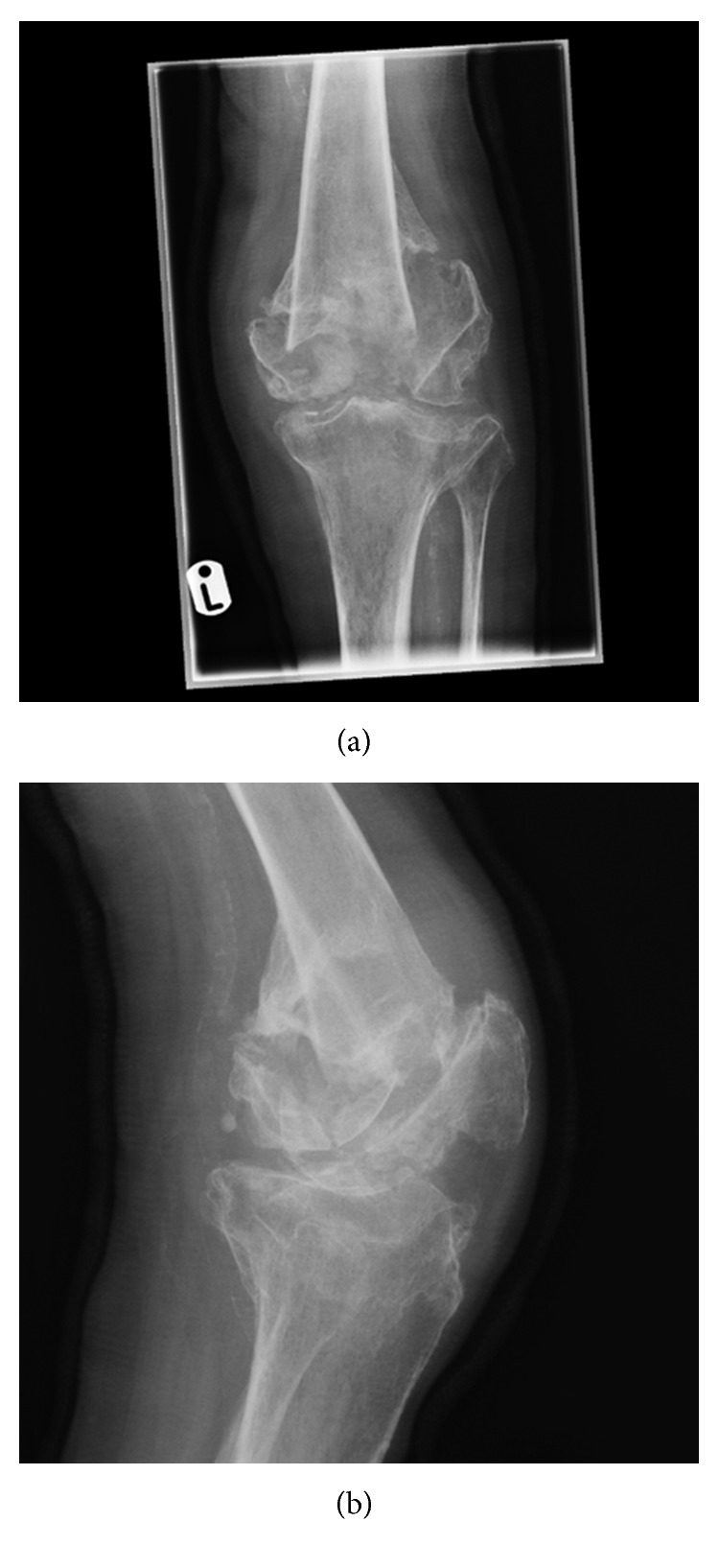
(a) AP and (b) lateral radiographs demonstrating ongoing nonunion and progressive deformity.

Despite conservative treatment, there were no radiological or clinical signs of union, and she developed progressive deformity (Figure 10) and a nontender pseudoarthrosis with ongoing instability. We discussed with the patient regarding open reduction and internal fixation or arthroplasty in the form of either a long-stemmed knee replacement or a distal femoral replacement. Amputation was also discussed: a decision by the patient and a review by the orthotist and the multidisciplinary team thought this not to be appropriate at this stage.

Radiologically the bone quality was poor. She is currently on a course of bisphosphonate treatment to improve this in order for further surgical treatment to go ahead.

## 4. Discussion

Diabetic neuroarthropathy involving large weight-bearing joints is rare, and the literature, although sparse, focuses on the complexities and the challenges of total knee arthroplasty in these patients.

Jean Martin Charcot's initial description of destructive hypertrophic arthropathy in 1886 [2] was in those patients with tabes dorsalis. Eichenholtz [3] took this further and classified the disease process into three stages: development, coalescence, and reconstruction. Patholophysiological mechanisms may include a combination of microtrauma (initial trauma with loss of neuroprotective mechanisms) and neurovascular (high-flow vascular state leading to osteopenia and deformity) processes [4].

### 4.1. Management of CK: Nonoperative

Nonoperative management has been described as far back as 1971 by Drennan et al. [5]. He recommended “complete rest” during acute episodes and pointed out that early return to activity carried a risk of “joint breakdown.” The difficulties with early recognition of acute Charcot were discussed in his small case series of 10 patients, demonstrating that the majority of patients presented with no or minimal pain despite gross deformity and instability.

Illgner et al. [1] describe three cases of CK and advocates conservative management due to the risk of postoperative complications following a total knee replacement (TKR) (prosthesis unknown) in two of their cases (ongoing bone loss and prosthesis dislocation). Jacquemin et al. [6] demonstrated successful nonoperative management with casting and non-weight bearing in a patient with CK. Initial X-rays showed minimal to moderate deformity.

Complete rest may not be appropriate if patients present with gross deformity. Miguel et al. [7] describe a case of CK where stability of a severely varus knee was attempted by external fixation for 5 months. Thereafter, there was eventual need for conversion to a full knee-ankle orthosis. Failure of conservative management was also seen by Lambert and Close [8] following plaster immobilisation for 3 months necessitating arthroplasty. The appropriate duration of immobilisation is controversial, with wide variations in the timing of this in the literature regarding foot and ankle Charcot being extrapolated to that of CK.

### 4.2. Management of CK: Arthrodesis

Early cases and their management are described as far back as 1931 by Cleveland and Smith [9] with four cases of postsyphilitic CK requiring arthrodesis. Modern prosthetic designs and techniques together with a younger patient cohort have meant that arthrodesis may now be a procedure of salvage rather than first choice. Fullerton and Browngoehl [10] describes bilateral CK of the knees, the first managed with arthrodesis to good clinical outcome.

### 4.3. Management of CK: Total Knee Arthroplasty

Although previously considered a relative contraindication for joint replacement, arthroplasty has seen resurgence with increasingly good clinical outcomes although challenges remain. Early cases were described as far back as the late 1980s and early 1990s [11–14]^.^

Kim et al. [15] describe a series of 19 knees in neurosyphilitic patients with Charcot arthropathy. The majority received cemented, constrained condylar prostheses. Over half (53%) had satisfactory outcomes, but a high proportion of complications was seen (47%). Their series highlights the challenges in CK and arthroplasty, with an initial uncemented and a second semiconstrained prostheses showing poor results before switching to a fully constrained option. The authors concluded fully constrained prostheses should be preferred although, together with long stems, this necessitates large amounts of bone stock to be removed, which may challenge revision cases. CK secondary to syphilis may do worse clinically than other pathologies.

Lambert and Close [8] describe a case of CK who failed conservative management with plaster immobilisation for three months who went on to have a TKA (prosthesis unknown). Good function four years postoperatively was reported.

A large case series of 40 TKRs in 29 patients sets out some important advice [16]. Diabetes only accounted for 7 of these replacements. The authors advocate that, despite the cause for the neuroarthropathy, TKR is a valid management. There were more complications in this group both intraoperatively (medial collateral ligament avulsion and tibial tubercle avulsion) and postoperatively (symptomatic instability, haematoma, and wound infection) when compared to nonneuropathic patients. Although gross range of motion and function was slightly inferior to those undergoing TKR for non-neuropathy knees, the majority of patients were symptom free in the long term. Of note, a high proportion of these patients required long-stemmed or constrained prostheses. The authors recommend selecting these in specific cases as well as careful attention to ligament balancing and augmentation of bony defects where appropriate.

Kucera et al. [17] describe a case of CK where cast immobilisation was not appropriate and early arthroplasty was performed with a simple posterior cruciate retaining prosthesis. Poor patient compliance led to early weight bearing and a radiolucent line developed under the tibial component. This was managed with a three-month period of non-weight bearing, and there had been no progression five months postoperatively.

Liu et al. [18] describe a case of bilateral neurosyphilitic Charcot knee. The right knee demonstrated deformity on plain radiographs, and due to immobilisation contraindications, a TKR was performed. The left knee had improved local symptoms with conservative management, but deformity had progressed forcing a TKR, again with a constrained rotational long-stemmed prosthesis.

Complications in this group of patients can be high and are multifactorial. Gross bone loss and synovitis present the orthopaedic surgeon with a hostile operating environment. Underlying pathology (e.g., ataxia or neurosyphilis) may complicate postoperative rehabilitation. Nakajima et al. [19] describe a case following TKR in a late presenter of amyloid Charcot neuropathy where extensive heterotopic ossification developed after the initial surgery. Using revision prostheses in the primary setting presents a challenge to future revision, with significant bone loss likely. Rates have been reported as high as 50% [20], but no distinction has been made as to whether this is higher in diabetics.

Our two cases had good clinical outcomes from periods of nonoperative management which was instilled by the multidisciplinary team that includes orthopaedic surgeons. It was possible to avoid surgical treatment in this series as the nonoperative measures, including offloading in a total contact cast and/or knee bracing and non-weight bearing, resulted in satisfactory outcomes. If recurrent episodes were to continue in these patients or structural integrity of the knee to be compromised with ongoing deformity or instability, an arthroplasty option would have been considered.

## 5. Conclusion

Charcot arthropathy of the knee is a rare and a possibly underdiagnosed complication of diabetes. It must be considered in diabetic patients who present with pain, swelling, or a warm knee. Our cases highlight that knee Charcot osteoarthropathy carries a higher risk of recurrence and concurrent bilateral pathology may occur. Prompt diagnosis and conservative management is appropriate in the acute phase to maintain structural integrity. Low-intensity pulsed ultrasound therapy was successfully used in one case. The medical literature describes a limited number of case reports and case series and suggests that future arthroplasty options are challenging to the orthopaedic surgeon once a change in morphology has occurred. Care must be taken in prosthesis choice, and there is no clear consensus. Decisions need to be made on a case-by-case basis. It is unclear whether the underlying pathology has an effect on longer term outcome. The increasing prevalence of diabetes may allow larger study cohorts in the future.
